# Efficacy of nano-modified Runji ointment in the treatment of mild and moderate psoriasis with blood dryness syndrome: a study protocol for a double-blind randomized controlled trial

**DOI:** 10.1097/MD.0000000000028178

**Published:** 2021-12-30

**Authors:** Guanru Li, Liyun Sun, Yue Qiu, Yaquan Hou, Libo Du, Kaixuan Zhao, Jiali Qian, Jiuli Liu, Tengfei Ma

**Affiliations:** a Department of Dermatology, Beijing Traditional Chinese Medicine Hospital Shunyi Hospital, Shunyi District, Beijing, China; b Department of Dermatology, Beijing Hospital of Traditional Chinese Medicine, Dongcheng District, Beijing, China; c Dongcheng District Yongdingmenwai Community Health Center, Beijing, China; d Institute of Chemistry, Chinese Academy of Sciences, Haidian District, Beijing, China; e Department of Dermatology, Handan Mingren Hospital, Hebei Province, China.

**Keywords:** modified Runji ointment, nano-sized, placebo, psoriasis, randomized controlled trial

## Abstract

**Introduction:**

Psoriasis is a common, recurrent, immune skin disease, which seriously affects patients’ quality of life. In clinical practice, modified Runji ointment can effectively treat mild-to-moderate psoriasis with blood dryness syndrome, but there is a lack of high-quality evidence-based medical evidence. This trial aims to evaluate the efficacy and safety of nano-modified Runji ointment in the treatment of mild-to-moderate psoriasis with blood dryness syndrome.

**Methods/design:**

This study will be a randomized double-blind placebo-controlled trial. A total of 80 patients will be recruited and randomly divided into an intervention group (nano-modified Runji ointment group) and a placebo group at a ratio of 1:1. All included patients will receive 8 weeks of nano-modified Runji ointment or placebo ointment respectively, twice a day. The primary outcome will be the change in psoriasis area and disease severity index score at week 8 compared to baseline. The secondary outcomes will be rash area score, pruritus score, Dermatology Life Quality Index score, traditional Chinese medicine symptom score and adverse events.

**Discussion:**

This study may provide high-quality evidence for the efficacy of nano-modified Runji ointment in the treatment of mild to moderate psoriasis with blood dryness syndrome. The results of this study will be published in peer-reviewed journals.

**Trial registration:**

ChiCTR, ChiCTR2000034292. Registered July 1, 2020, https://www.chictr.org.cn/edit.aspx?pid=55884&htm=4

## 1 Introduction

Psoriasis is a common chronic inflammatory skin disease.^[1]^ Epidemiology surveys worldwide have found that its incidence is about 2%.[^2^,^3^] Most scholars believe that psoriasis is an inflammatory response caused by an abnormal immune system,[^4^,^5^] accompanied by a variety of skin rashes.^[6]^ Psoriasis vulgaris is the most common type of psoriasis.^[7]^ The pathological feature of psoriasis vulgaris is continuous inflammation, leading to uncontrolled proliferation and dysfunctional differentiation of keratinocytes.[^8^,^9^] Common symptoms of psoriasis vulgaris include itching, burning, and pain.^[10]^ The severity of rash was reversible. Most patients with psoriasis vulgaris exhibit recurrence characteristics. Within a few weeks or months, the number and area of rashes increase rapidly.^[11]^

For patients with mild to moderate psoriasis, most of the current diagnosis and treatment guidelines recommend the use of topical medicine.[^12^,^13^] Topically applied medicines can be absorbed through the skin into local blood microcirculation and directly act on skin lesions. At the same time, the first-pass effect of the liver and gastrointestinal inactivation of oral administration can be avoided. The constant blood concentration or physiological effects of the skin lesion can be maintained, and the action time can be prolonged. The most common topically applied medicines are glucocorticoids, tretinoin, vitamin D3 derivatives, and other drugs. Topically applied medicines have relatively satisfactory clinical effects. However, long-term use of glucocorticoid drugs can cause skin atrophy, telangiectasia, hirsutism, etc^[14]^; use of tretinoin drugs can cause irritant dermatitis, photosensitivity, etc[^15^,^16^]; some patients use vitamin D derivatives with side effects such as skin irritation and burning sensation.^[13]^

In clinical practice, external treatment with traditional Chinese medicine (TCM) also has a positive effect. Runji ointment is a common medicine used to treat psoriasis with blood dryness syndrome by ancient TCM doc. Our pre-experiment showed that modified Runji ointment can alleviate the symptoms of dry skin rash and itching in patients with psoriasis of blood dryness syndrome, and promote the regression of the rash.^[17]^ However, the particle diameter of TCM preparations is relatively large, which affects the transdermal absorption rate of the medicine. In recent years, some scholars have proposed nano-drug delivery systems (NDDS),[^18^,^19^] that is, by NDDS,^[20]^ the particle radius of the drug is smaller, and the surface area is larger. The drug's adhesiveness is improved, thereby improving the bioavailability of the drug; and nano-drugs can pass through some physical barriers and have a wider distribution range, which enhances drug utilization.^[21]^ The objective of this study was to evaluate the efficacy and safety of nano-modified Runji ointment compared with placebo ointment in the treatment of mild to moderate psoriasis with blood dryness syndrome.

## 2 Registration and ethics

Before the first patient was enrolled in this study, it was registered with the Chinese Clinical Trial Registry, registration no. ChiCTR2000034292 (2020/07/01. http://www.chictr.org.cn/edit.aspx?pid=55884&htm=4). This study was conducted in accordance with the principles of the Declaration of Helsinki (2014 edition). The protocol was reviewed and approved by the Medical Ethics Committee of Shunyi Hospital of Beijing Traditional Chinese Medicine Hospital (approval number 2020SYKY01–02, protocol version number: 2nd version, version date: April 19, 2020).

## 3 Patient recruitment and informed consent

This study will be a multicenter randomized double-blind placebo-controlled trial, including an 8-week treatment period and a 16-week follow-up period. The study design will follow the CONSORT Extension for Chinese Herbal Medicine Formulas 2017: Recommendations, Explanation, and Elaboration^[22]^ for reporting. A total of 80 participants will be recruited from the Dermatology Clinics of Shunyi Hospital of Traditional Chinese Medicine and Beijing Hospital of Traditional Chinese Medicine. Before enrollment, the principal investigators of each center will introduce the potential benefits and risks of the study to the participants. All participants will sign an informed consent form before being enrolled in the study.

## 4 Inclusion criteria

The inclusion criteria were as follows: meeting the diagnostic criteria for Western medicine psoriasis vulgaris; meeting the syndrome differentiation standard for psoriasis with blood dryness syndrome; aged 18 to 65 years; mild or moderate degree of skin lesions (skin area < 30% of body surface area); and signed the informed consent form. The informed consent process was in accordance with the provisions of good clinical practice.

## 5 Exclusion criteria

Any of the following cases will be excluded: patients treated with vitamin D 3, retinoic acid, or corticosteroids within 1 month; those who are allergic to the study drug; complicated with cardiovascular, cerebrovascular, liver, kidney, hematopoietic system, and other serious primary diseases and mental patients; pregnant or lactating women with family planning within 3 months; and patients who are participating in clinical trials with other drugs.

## 6 Randomization

The random sequence will be generated by the professionals of the data management center using SAS 9.2 software (SAS Institute Inc., Cary, NC). According to the ratio of 1:1, patients will be assigned to the intervention group (nano-modified Runji ointment group) and placebo group. The random allocation table and random code will be kept strictly confidential, and the allocation hiding will continue throughout the experiment.

## 7 Blinding

The treatment plan will blind the enrolled patients, and the investigator and clinical pharmacist will also be blinded. Only the Key Unit of Methodology in Clinical Research of Shunyi Hospital of Traditional Chinese Medicine has access to the random allocation table. Those who participated in the study and those related to the trial will also be blinded. After the study is completed, the research team can view the blind assignment. In the event of a medical emergency, it is necessary to understand the patient's previous treatment and the current treatment plan, and the researcher will unblind. The researcher will try to contact the project leader first, then contact the relevant statistician, and use a separately arranged emergency code to unblind.

## 8 Intervention

All enrolled patients underwent the TCM decoction Yangxue Huoxue Decoction, a prescription for the treatment of psoriasis with blood dryness syndrome. It is composed of *Radix Angelicae sinensis, Caulis Spatholobi, Rhizoma Dioscoreae, Radix Rehmanniae recens, Rhizoma Smilacis Glabrae, Nidus Vespae,* and *Radix Clematidis*. The drug dosage form was granules, twice a day for 8 weeks, and it is provided by Guangdong Yifang Pharmaceutical Co., Ltd.

The medicine used by the intervention group is nano-modified Runji ointment. Its ingredients are Radix *A sinensis*, Fructus Cannabis, *Caulis* Spatholobi, *Caulis* Polygoni Multiflori, Fructus Aurantii, Radix *Angelicae dahuricae*, Radix *Clematidis*, and Radix Lithospermi. Preparation process: these medicines were soaked in sesame oil for 48 hours, and then fried at an oil temperature of 100°C to 120°C until they were browned, the oil was filtered, and then the medicines were nanometerized using nanotechnology to make nano ointment. The placebo ointment used by the placebo group is mainly composed of petrolatum and pigment, which makes its properties and appearance look similar to the ointment in the intervention group. The ointments for the intervention and placebo groups were obtained from the Institute of Chemistry, Chinese Academy of Sciences.

**Figure 1:**
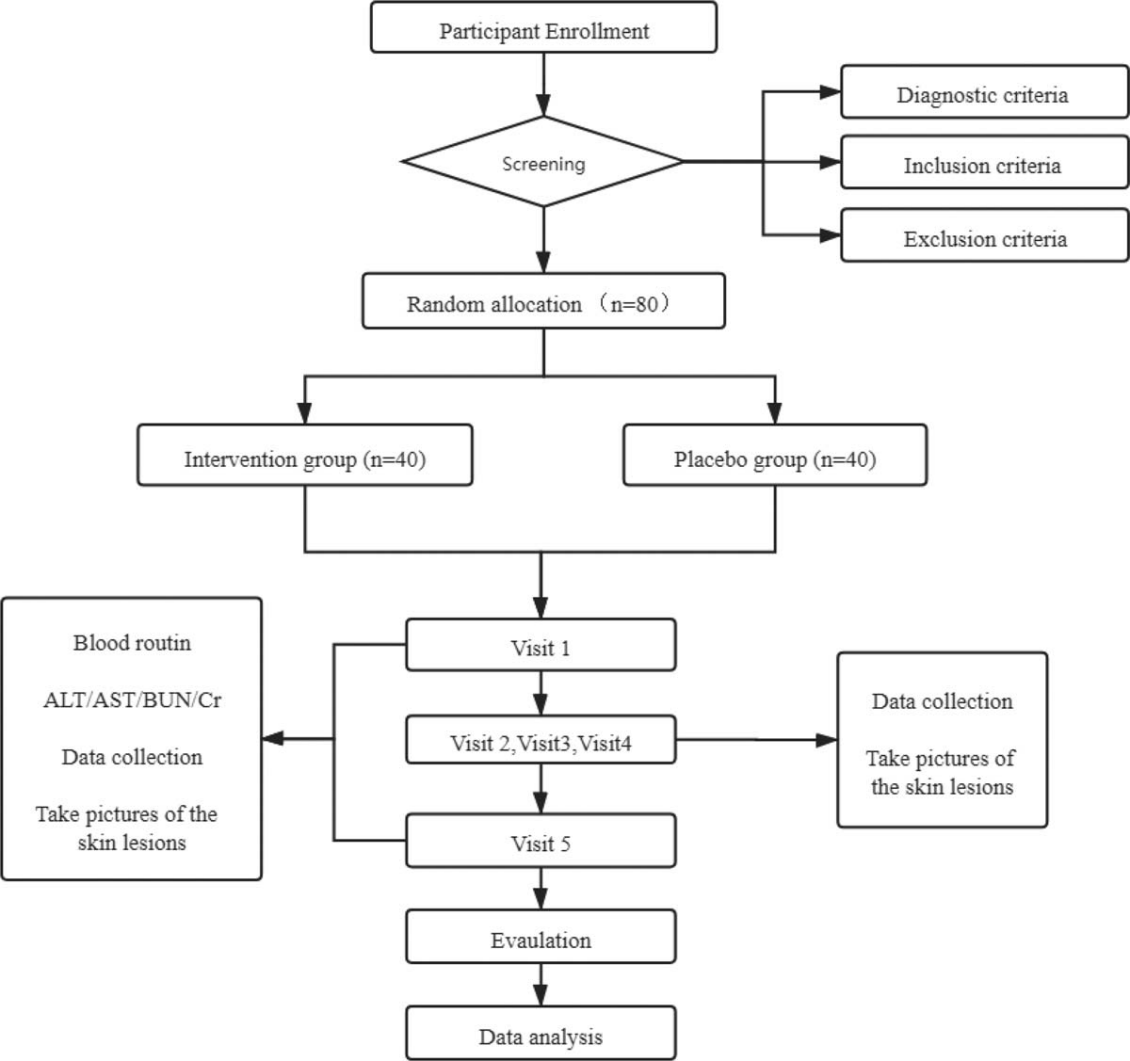
The flow chart of the trial. ALT = alanine aminotransferase, AST = aspartate aminotransferase, BUN = blood urea nitrogen, Cr = serum creatinine.

The frequency of use of the nano-modified Runji ointment and placebo ointment was twice a day for 8 weeks. The dosage of each drug was determined according to the size of the skin lesions. Approximately 2 palm areas (approximately 2% of the body surface area) can use 1 fingertip unit ointment.^[23]^ One fingertip unit refers to the amount of ointment covered from the tip of the index finger to the first interphalangeal joint with a tube with a diameter of 5 mm. The flow chart of the trial is shown in Figure 1.

## 9 Outcomes

### 9.1 Primary outcome

The primary outcome was the change in the psoriasis area and disease severity index score (PASI) from baseline to week 8. PASI is the gold standard for the diagnosis and evaluation of psoriasis, a simple and effective scale proposed in 1978.[^24^,^25^] The calculation method is as follows:


$$\text{PAS}{\text{I}}_{\text{H}}=({\text{E}}_{\text{H}}+{\text{I}}_{\text{H}}+{\text{D}}_{\text{H}})\times {\text{A}}_{\text{H}}\times 0.1$$

$$\text{PAS}{\text{I}}_{\text{U}}=({\text{E}}_{\text{U}}+{\text{I}}_{\text{U}}+{\text{D}}_{\text{U}})\times {\text{A}}_{\text{U}}\times 0.2$$

$$\text{PAS}{\text{I}}_{\text{L}}=({\text{E}}_{\text{L}}+{\text{I}}_{\text{L}}+{\text{D}}_{\text{L}})\times {\text{A}}_{\text{L}}\times 0.4$$

$$\text{PASI}=\text{PAS}{\text{I}}_{\text{H}}+\text{PAS}{\text{I}}_{\text{U}}+\text{PAS}{\text{I}}_{\text{T}}+\text{PAS}{\text{I}}_{\text{L}}$$

E, erythema; I, infiltration; D, scales; A, area; H, head; U, upper limbs; T, trunk; L, lower limbs. The scoring range of skin lesion area: 1, <10%; 2, 10% to 30%; 3, 30% to 50%; 4, 50% to 70%; 5, 70% to 90%; 6, 90% to 100%. The scores for I and D ranged from 0 to 4: 0, no symptom; 1, mild; 2, moderate; 3, severe; 4, very severe. E's scoring method: 0, no erythema; 1, mild erythema; 2, moderate erythema; 3, significant erythema; 4, severe erythema. The total PASI ranged from 0 to 72 points. The higher the score, the more serious the condition.

### 9.2 Secondary outcome

#### 9.2.1 Body surface area score

The area of skin lesions was evaluated using the palm area method.^[26]^ The fully extended area of the subject's palm represented 1% of the body surface area.

#### 9.2.2 Visual analogue scale method

The visual analog scale method was used to evaluate the degree of itching of the subjects in the past week, which is represented by a 100 mm long line. 0: no itching, 100 mm: most itching, and the degree of itching is unbearable.

#### 9.2.3 Dermatology Life Quality Index score

The Dermatology Life Quality Index is a questionnaire commonly used in the daily diagnosis and treatment of the Dermatology Department.^[27]^ It contains 10 questions to evaluate the impact of skin diseases on the quality of life. The score ranges from 0 to 30 points; the higher the score, the lower the quality of life.

#### 9.2.4 TCM symptom score

The TCM symptom score was used to measure changes in the symptoms of blood dryness syndrome. The syndrome score scale was divided into 3 dimensions: scaly, itching, and dry mouth. The score of each dimension was 0 to 7 points, and the total score was 0 to 21 points. The higher the score, the more severe the symptoms.

All patients will be followed up by experienced investigators (≥5 years of clinical experience). The above outcome indicators will be evaluated at 0, 2, 4, 6, 8, 12, and 24 weeks. All the observed indicators are shown in Table 1.

**Table 1:** Schedule for enrollment, intervention, and assessment.

Factors	Screening	Treatment period				Follow-up	
	Visit 1	Visit 2	Visit 3	Visit 4	Visit 5	Visit 6	Visit 7
	Week 0	Week 2	Week 4	Week 6	Week 8	Week 12	Week 24
Informed consent	√						
Enrollment	√						
Demographic history	√						
Medical history	√						
Physical examinations	√						
Conformation of inclusion and exclusion criteria	√						
Randomization	√						
Medication	√						
Safety index (blood routine examination, ALT/AST/BUN/Cr)	√				√		
AE		√	√	√	√	√	√
PASI/VAS/BSA score	√	√	√	√	√	√	√
TCM symptom score	√	√	√	√	√	√	√
DLQI	√				√	√	√
Take pictures of the lesions	√	√	√	√	√	√	√

#### 9.2.5 Adverse events (AEs)

All adverse events (AEs) will be recorded and any serious AEs will be reported to the Research Ethics Committee within 24 hours. When AEs occur, the study investigators will request that the patient terminate the treatment and then determine whether this event was related to the nano-modified Runji ointment. The principal investigator may implement urgent safety measures to protect patients from immediate harm. If the AE is related to the nano-modified Runji ointment, it is termed as a side effect. If the side effect is mild and the patient agrees to commence, the treatment will continue after the symptoms abate or disappear. Patients will withdraw from the study if serious AEs are observed.

### 9.3 Sample size estimation

We expect that after the end of the treatment period, the PASI of patients in the intervention group will drop by 2 more than in the placebo group, with a standard deviation of 3. It is now inferred that the probability of type I error is 5% on both sides, and the probability of type II error is 20%. Therefore, 36 patients were included in the study. Considering the 10% loss to follow-up rate, each group needs to recruit 40 patients.

### 9.4 Data collection and management

Data collection and management are required before the start, during, after treatment, and during the follow-up period. Research data was recorded on the case report form form and then entered into the electronic system (double data entry). All personal private information about the patients was hidden.

### 9.5 Statistical analysis

A statistician blinded to the patient allocation for each group performed all analyses using SPSS 21.0 software (SPSS Inc, Chicago, IL). Statistical analysis will be performed on an intention-to-treat basis. The intention-to-treat analysis included all randomized patients. We will describe the data using frequency counts and proportions for qualitative variables and means ± standard deviation will be used for normally distributed quantitative variables, and median (interquartile) will be used for non-normally distributed quantitative variables. We will apply the chi-square test to examine the differences between the intervention and placebo groups for qualitative variables, the t-test to examine the differences between 2 groups for normally distributed quantitative variables, and the Wilcoxon rank-sum test for non-normally distributed quantitative variables. A generalized linear model was applied to quantitative variables with repeated measures. In this study, *P* values <.05 (two-tailed) will be considered statistical significance.

## 10 Discussion

The WHO regards psoriasis as a serious global problem.^[28]^ Topically applied medicines have become the focus of many current studies. The purpose of this study was to evaluate the efficacy and safety of nano-modified Runji ointment in the treatment of mild-to-moderate psoriasis with blood dryness syndrome.

TCM has a long history of treating psoriasis, which divides psoriasis into 3 types of syndromes: blood heat syndrome, blood stasis syndrome, and blood dryness syndrome.[^29^,^30^] For the treatment of blood heat syndrome and blood stasis syndrome, many researchers have adopted a variety of research methods such as oral Chinese herbal decoctions,^[31]^ topically applied Chinese herbal ointments,^[32]^ and application of acupuncture and moxibustion.^[33]^ After the treatment, the skin lesion area, degree of infiltration, and degree of pruritus of patients are significantly improved compared to before the treatment. The quality of life significantly improved, and the curative effect was satisfactory. There are many patients with psoriasis of blood dryness syndrome, but there are few related clinical studies. Therefore, the focus of this study was the treatment of patients with blood dryness syndrome.

Runji ointment, from *Golden Mirror of Medicine*, is a topically applied ointment used by ancient TCM doctor to treat psoriasis. It consists of *Radix A sinensis* and *Radix Lithospermi*. According to the clinical manifestations of patients with blood dryness syndrome, our research group added *Fruits Cannabis, Caulis Spatholobi, Caulis Polygoni Multiflori, Fructus Aurantii, Radix A dahuricae,* and *Radix Clematidis*, and made them into modified Runji ointment. Previous studies have shown that the modified Runji ointment can effectively alleviate the symptoms of psoriasis with blood dryness syndrome.^[17]^ However, some patients said that the absorption of the drug was not good, and the comfort during use was poor. This may be due to the fact that the traditional preparation method of modified Runji ointment has a large particle diameter and is hindered by the skin barrier, resulting in a low transdermal absorption rate of the drug. Therefore, in this study, we used nanotechnology to nanometerize the drug into a nano-modified Runji ointment to allow it to smoothly pass through the skin barrier. We hope that this method can increase the absorption rate of topically applied drugs and improve the curative effect. At the same time, the dosage of the drug is reduced and safety is improved.

However, this study also has some limitations: first, this study only included patients with mild-to-moderate blood dryness psoriasis. It is not clear whether the modified psoriasis is effective in patients with severe psoriasis and other syndromes. Second, the ointment is taken home by the patient for self-use. Although the patient was required to record the medication events and conditions, the true medication compliance could not be confirmed.

## Author contributions

**Conceptualization:** Guanru Li, Liyun Sun.

**Data curation:** Guanru Li.

**Funding acquisition:** Guanru Li.

**Investigation:** Yue Qiu, Libo Du, JiaLi Qian, JiuLi Liu, TengFei Ma.

**Methodology:** Guanru Li, YaQuan Hou, Liyun Sun.

**Project administration:** Guanru Li.

**Resources:** Libo Du, Kaixuan Zhao.

**Writing - original draft:** Guanru Li.

**Writing - review & editing:** Guanru Li, Liyun Sun.

## References

[1] Nast A Jacobs A Rosumeck S Werner RN. Methods report: European S3-guidelines on the systemic treatment of psoriasis vulgaris – update 2015 – EDF in cooperation with EADV and IPC. J Eur Acad Dermatol Venereol 2015;29:e1–22. 10.1111/jdv.13353 26471228

[2] Parisi R Symmons DP Griffiths CE Ashcroft DM. Global epidemiology of psoriasis: a systematic review of incidence and prevalence. J Invest Dermatol 2013;133:377–85. 23014338 10.1038/jid.2012.339

[3] Greb JE Goldminz AM Elder JT. Psoriasis. Nat Rev Dis Primers 2016;2:16082. 27883001 10.1038/nrdp.2016.82

[4] Ogdie A Gelfand JM. Clinical risk factors for the development of psoriatic arthritis among patients with psoriasis: a review of available evidence. Curr Rheumatol Rep 2015;17:64. 26290111 10.1007/s11926-015-0540-1 PMC5278907

[5] Kamiya K Kishimoto M Sugai J Komine M Ohtsuki M. Risk factors for the development of psoriasis. Int J Mol Sci 2019;20:18. 10.3390/ijms20184347 PMC6769762 31491865

[6] McGraw-Hill, Cox NH Cox NH. Fitzpatrick's Dermatology in General Medicine. 2012;197–231. Health Professions Division.

[7] Lebwohl M. Psoriasis. Lancet 2003;361:1197–204. 12686053 10.1016/S0140-6736(03)12954-6

[8] Griffiths CE Barker JN. Pathogenesis and clinical features of psoriasis. Lancet 2007;370:263–71. 17658397 10.1016/S0140-6736(07)61128-3

[9] Rendon A Schäkel K. Psoriasis pathogenesis and treatment. Int J Mol Sci 2019;20:1475. 10.3390/ijms20061475 PMC6471628 30909615

[10] Weigle N McBane S. Psoriasis. Am Fam Physician 2013;87:626–33. 23668525

[11] Raychaudhuri SK Maverakis E Raychaudhuri SP. Diagnosis and classification of psoriasis. Autoimmun Rev 2014;13:490–5. 24434359 10.1016/j.autrev.2014.01.008

[12] Nast A Smith C Spuls PI. EuroGuiDerm guideline on the systemic treatment of psoriasis vulgaris – part 1: treatment and monitoring recommendations. J Eur Acad Dermatol Venereol 2020;34:2461–98. 33349983 10.1111/jdv.16915

[13] Menter A Korman NJ Elmets CA. Guidelines of care for the management of psoriasis and psoriatic arthritis. Section 3. Guidelines of care for the management and treatment of psoriasis with topical therapies. J Am Acad Dermatol 2009;60:643–59. 19217694 10.1016/j.jaad.2008.12.032

[14] Hengge UR Ruzicka T Schwartz RA Cork MJ. Adverse effects of topical glucocorticosteroids. J Am Acad Dermatol 2006;54:01–15. quiz 16–8. 10.1016/j.jaad.2005.01.010 16384751

[15] Mukherjee S Date A Patravale V Korting HC Roeder A Weindl G. Retinoids in the treatment of skin aging: an overview of clinical efficacy and safety. Clin Interv Aging 2006;1:327–48. 18046911 10.2147/ciia.2006.1.4.327 PMC2699641

[16] Carrascosa JM Vanaclocha F Borrego L. Update of the topical treatment of psoriasis. Actas Dermosifiliogr 2009;100:190–200. 19457304

[17] Guanru L. Liyun S. Beijing University of Chinese Medicine, Clinical Study on Modified Runji Ointment in the Treatment of Psoriasis with Blood Dryness Syndrome Based on Prescrption with “Analogous Selection of Herbs”. Beijing:2017.

[18] Zhou F Teng F Deng P Meng N Song Z Feng R. Recent progress of nano-drug delivery system for liver cancer treatment. Anticancer Agents Med Chem 2018;17:1884–97. 28707574 10.2174/1871520617666170713151149

[19] Wang W Lu KJ Yu CH Huang QL Du YZ. Nano-drug delivery systems in wound treatment and skin regeneration. J Nanobiotechnol 2019;17:82. 10.1186/s12951-019-0514-y PMC6617859 31291960

[20] Huang Y Zhao Y Liu F Liu S. Nano traditional Chinese medicine: current progresses and future challenges. Curr Drug Targets 2015;16:1548–62. 25751006 10.2174/1389450116666150309122334

[21] Bhattacharya D Ghosh B Mukhopadhyay M. Development of nanotechnology for advancement and application in wound healing: a review. IET Nanobiotechnol 2019;13:778–85. 31625517 10.1049/iet-nbt.2018.5312 PMC8676206

[22] Cheng CW Wu TX Shang HC. CONSORT extension for Chinese herbal medicine formulas 2017: recommendations, explanation, and elaboration. Ann Intern Med 2017;167:112–21. 28654980 10.7326/M16-2977

[23] Dekio I Morita E. The weight of a finger-tip unit of ointment in 5-gram tubes. J Dermatolog Treat 2011;22:302–3. 20673156 10.3109/09546631003797098

[24] Schmitt J Wozel G. The psoriasis area and severity index is the adequate criterion to define severity in chronic plaque-type psoriasis. Dermatology 2005;210:194–9. 15785046 10.1159/000083509

[25] Fredriksson T Pettersson U. Severe psoriasis – oral therapy with a new retinoid. Dermatologica 1978;157:238–44. 357213 10.1159/000250839

[26] Mosteller RD. Simplified calculation of body-surface area. N Engl J Med 1987;317:1098. 3657876 10.1056/NEJM198710223171717

[27] Finlay AY Khan GK. Dermatology Life Quality Index (DLQI) – a simple practical measure for routine clinical use. Clin Exp Dermatol 1994;19:210–6. 8033378 10.1111/j.1365-2230.1994.tb01167.x

[28] Michalek IM Loring B John SM. A systematic review of worldwide epidemiology of psoriasis. J Eur Acad Dermatol Venereol 2017;31:205–12. 27573025 10.1111/jdv.13854

[29] Zhang GZ Wang JS Wang P. Distribution and development of the TCM syndromes in psoriasis vulgaris. J Tradit Chin Med 2009;29:195–200. 19894384 10.1016/s0254-6272(09)60064-9

[30] Meng S Lin Z Wang Y Wang Z Li P Zheng Y. Psoriasis therapy by Chinese medicine and modern agents. Chin Med 2018;13:16. 29588654 10.1186/s13020-018-0174-0 PMC5865286

[31] Chen X Zhang R Duan X Xue M Qu T Li L. Effectiveness of Xiaoyin Jiedu granules in the treatment of psoriasis vulgaris in patients with blood-heat symptom patterns in terms of traditional Chinese medicine. J Tradit Chin Med 2020;40:863–9. 33000588 10.19852/j.cnki.jtcm.2020.05.017

[32] Li N Zhao W Xing J. Chinese herbal Pulian ointment in treating psoriasis vulgaris of blood-heat syndrome: a multi-center, double-blind, randomized, placebo-controlled trial. BMC Complement Altern Med 2017;17:264. 28506228 10.1186/s12906-017-1631-5 PMC5432985

[33] Liu L Lu Y Yan XN. Efficacy and safety of fire needle therapy for blood stasis syndrome of plaque psoriasis: protocol for a randomized, single-blind, multicenter clinical trial. Trials 2020;21:739. 32843084 10.1186/s13063-020-04691-7 PMC7446129

